# Cyclic mechanical strain with high-tensile triggers autophagy in growth plate chondrocytes

**DOI:** 10.1186/s13018-022-03081-w

**Published:** 2022-03-28

**Authors:** Jin-ming Zhang, Zheng-gang Wang, Zhi-yi He, Liang Qin, Jiang Wang, Wen-tao Zhu, Jun Qi

**Affiliations:** Department of Orthopedics, Tongji Hospital, Tongji Medical College, Huazhong University of Science and Technology, Wuhan, 430030 Hubei Province People’s Republic of China

**Keywords:** Cyclic tensile strain, Chondrocytes, Autophagy, Cytochalasin D

## Abstract

**Background:**

Mechanical loading has been widely considered to be essential for growth plate to maintain metabolism and development. Cyclic mechanical strain has been demonstrated to induce autophagy, whereas the relationship between cyclic tensile strain (CTS) and autophagy in growth plate chondrocytes (GPCs) is not clear. The objective of this study was to investigate whether CTS can regulate autophagy in GPCs in vitro and explore the potential mechanisms of this regulation.

**Methods:**

The 2-week-old Sprague–Dawley rat GPCs were subjected to CTS of varying magnitude and duration at a frequency of 2.0 Hz. The mRNA levels of autophagy-related genes were measured by RT-qPCR. The autophagy in GPCs was verified by transmission electron microscopy (TME), immunofluorescence and Western blotting. The fluorescence-activated cell sorting (FACS) was employed to detect the percentage of apoptotic and necrotic cells.

**Results:**

In GPCs, CTS significantly increased the mRNA and protein levels of autophagy-related genes, such as *LC3*, *ULK1*, *ATG5* and *BECN1* in a magnitude- and time-dependent manner. There was no significant difference in the proportion of apoptotic and necrotic cells between control group and CTS group. The autophagy inhibitors, 3-methyladenine (3MA) and chloroquine (CQ) reversed the CTS-induced autophagy via promoting the formation of autophagosomes. Cytochalasin D (cytoD), an inhibitor of G-actin polymerization into F-actin, could effectively block the CTS-induced autophagy in GPCs.

**Conclusion:**

Cyclic mechanical strain with high-tensile triggers autophagy in GPCs, which can be suppressed by 3MA and CQ, and cytoskeletal F-actin microfilaments organization plays a key role in chondrocytes’ response to mechanical loading.

## Introduction

The epiphysis growth plate is a transient form of cartilage and a necessary structure for longitudinal bone growth. It is well known that chondrocytes are embedded within the abundant extracellular matrix (ECM) in cartilage. Previous studies have reported that mechanical loading is required to regulate growth plate function, especially the proliferation of chondrocytes [1, 2]. Under physiological conditions, the growth plate receives compression loading from articular surfaces and stretch loading from tendons and ligaments during limb movement, resulting in deformation and flow of ECM around chondrocytes [3]. Thus, chondrocytes in ECM are subjected to biomechanical loadings such as compressive stress, tensile stress and shear stress [4]. Mechanical stimulation is important for skeletal integrity, and it has been clearly established that routine exercise is essential to maintain normal bone mass [5]. When the epiphysis is in an abnormal biomechanical microenvironment (such as gonyectyposis, gonycrotesis and idiopathic scoliosis), bone grows may be accompanied with ecchymosis and/or disorders, leading to deformity and disability [6, 7]. Meanwhile, mechanical force is essential for chondrocytes maintenance of metabolic processes and healthy composition of cartilage ECM [8]. Previous research showed that appropriate cyclic tensile strain (CTS), with 3000 µs at a frequency of 0.5 Hz, can regulate proliferation of growth plate chondrocytes via ERK and YAP signal pathway [9, 10]. However, Villemure et al. reported that a 2-Hz Haversian wave with peak loads of 17 N might damage the structure of articular cartilage and as a consequence, its function [11].

Autophagy is a conservative and ubiquitous physiological process that triggered by hypoxia, oxidative stress, nutrient deprivation and other cellular stress to maintain cellular homeostasis and promote cell survival and adaptation to unamiable microenvironment [12]. Although autophagy is typically a nonselective response to starvation and other stresses, the process also occurs under nutrient-rich conditions (referred to as basal autophagy or constitutive autophagy), which prevents the accumulation of disease-related proteins [13]. Cinque et al. found that autophagy is involved in the development of growth plate and can regulate the synthesis of type II collagen [14]. In another study, the result suggested that autophagy takes part in the maturation and the terminal differentiation of chondrocytes, as well as their survival under stress and hypoxia conditions [15]. Previous studies have indicated that autophagy protects growth plate chondrocytes (GPCs) from intermittent CTS-induced damage [16, 17]. There are accumulating researches that have pointed that mechanical loading can regulate the level of autophagy [18, 19]. Lin et al. found that the autophagy level of cardiomyocytes increased after the intervention by tensile strain [20]. However, the relationship of autophagy and CTS in GPCs is unclear. Therefore, to explore the relationship between biomechanical signals and chondrocyte autophagy, find the appropriate equilibrium point and study the mechanism behind it and extend the results to in vitro and in vivo research models, this is of great significance for basic research and clinical transformation.

In addition, previously studies have shown a critical function for cytoskeletal elements in the biological process of autophagy [21, 22]. Mechanical signals are transmitted into cells through the ECM-integrin signaling pathway and transferred into nucleus under the action of cytoskeleton [23–25]. Besides, cytoskeleton elements are involved in numerous physiological processes such as mitosis, cell motility, membrane trafficking, body growth and development [19, 26]. The cytoskeleton of a cell is composed of actin, microtubules, intermediate filaments and septins. Actin is a globular, multifunctional protein that forms filaments. The monomeric form is called globular actin (G-actin) and polymerized form is known as filamentous actin (F-actin), which can be inhibited by cytochalasin D (cytoD) [27, 28].

Here we applied the four-point bending system to investigate whether autophagy could be induced by the different magnitude of CTS in rat GPCs and to explore the role of F-actin in CTS-induced GPCs autophagy. The results of this study can be extended to in vitro and in vivo models for preclinical research and a new sight that CTS may maintain GPCs homeostasis by mediating autophagy.

## Methods

### Antibodies and reagents

The following antibodies were used for Western blotting: microtubule-associated protein 1 light chain 3 A/B (LC3A/B, #12741, dilution 1:1000), autophagy-related 5 (ATG5, #12994S, dilution 1:1000), Beclin-1 (BECN1, #3495s, dilution 1:1000), unc-51-like autophagy-activating kinase 1 (Ulk-1, #8054S, dilution 1:1000), B-cell lymphoma-2 (Bcl-2, #2870s, dilution 1:1000) and sequestosome 1/p62 (SQSTM1/p62, #5114s, dilution 1:1000), purchased from Cell Signaling Technology (CST, Danvers, MA, USA). Glyceraldehyde-3-phosphate dehydrogenase (GAPDH, PB0141, dilution 1:400) and horseradish peroxidase (HRP)-labeled goat-anti-rabbit antibody (BA1055, dilution 1:500) were purchased from Boster (Wuhan, Hubei, China). For immunofluorescence (IF) detection, anti- LC3A/B (dilution 1:100) was obtained from CST, Rabbit IgG Alexa fluor-594 (dilution 1:200) was obtained from Invitrogen (Grand Island, NY, USA), Actin-tracker Green (dilution 1:200) and 4',6-diamidino-2-phenylindole dihydrochloride (DAPI, C1002, 1 μg/mL) were purchased from Beyotime (Shenzhen, Guangdong, China). For flow cytometric analysis, Annexin V/ propidium iodide (PI) apoptosis kit was obtained from MultiSciences Biotech (Hangzhou, Zhejiang, China). For autophagy flux studies, 3-methyladenine (3MA) and chloroquine (CQ) were purchased from Selleck Chemicals (Houston, TX, USA). To inhibit polymerization into F-actin, cytoD was obtained from Sigma-Aldrich (St. Louis, MO, USA).

### GPCs isolation and culture

GPCs were obtained from 2-week-old Sprague–Dawley rats. All animal procedures were approved by the Ethical Committee of Tongji Hospital, Tongji Medical College, Huazhong University of Science and Technology (Wuhan, Hubei, China). As described in previously published study [9], the skin and soft tissues surrounding the distal femur and proximal tibia were snipped sufficiently, and the epiphyseal plate cartilage was separated and cut into tiny fragments under an operating microscope. After rinsed in phosphate-buffered saline (PBS) several times, the fragments were digested with 0.25% trypsin solution (Sigma-Aldrich, USA) and 0.2% type II collagenase solution (Sigma-Aldrich, USA) at 37.0 °C for 30 min and 6 h, respectively. The obtained chondrocytes were collected, centrifuged and resuspended in Dulbecco's modified Eagle's medium/Ham's F-12 (DMEM/F-12, HyClone, USA) containing 10% fetal bovine serum (FBS, Gibco, USA) and antibiotics (100 U/mL penicillin G sodium, 100 μg/mL streptomycin sulfate and 0.5 μg/mL Fungizone). Cells were maintained at 37.0 °C in the incubator with 5% CO_2_ and were subcultivated 1:3 when 80% confluent. To preserve the chondrocyte phenotype, only cells of the third passage were used for subsequent experiments.

### Application of cyclic mechanical strain

The detailed methods and procedure have been described in a previous study [9]. CTS was performed via the four-point bending system, the theories and details of which have been described by Cai et al. [29]. The device can be placed in general incubator to maintain at 37 °C with 5% CO_2_ during loading experiments [30]. Before seeding cells, dishes for mechanical loading were autoclaved and culture plates sterilized with ethylene oxide. After rinsed in PBS, culture plates were coated with fibronectin solution (100 μg/mL in PBS) overnight. The sorted chondrocytes were seeded onto culture plates (size 78 mm × 38 mm) at a density of 1.5 × 10^5^ cells per plate and incubated at 37.0 °C for 24 h to permit GPCs adhering to the surface completely. Culture plates that GPCs attached were laid inside of the bending dishes and the medium were replaced with serum-free medium. Then the chondrocytes were exposed to CTS at a frequency of 2.0 Hz with different magnitude (1000 με, 2500 με, 4000 με and 5500 με) and time (0.5 h, 2 h and 6 h). Before mechanical stimulation, 3MA (10 mM), CQ (50 μM) or cytoD (1 μM) was added into the medium and incubated for 1 h. As a control, GPCs cultured on another plate were kept in the same dish under the test plate, but not subjected to mechanical loading for the same period.

### RNA extraction and real-time RT-PCR analysis

RNA extraction from GPCs was performed using E.Z.N.A. Total RNA Kit I (Omega Bio-Tek, Norcross, GA, USA) according to the manufacturer's instructions and reverse transcription-quantitative polymerase chain reaction (RT-qPCR) protocols were as described [31]. In brief, the purified RNA was eluted with RNase-free water and retro-transcribed using ReverTra Ace qPCR RT kit (Toyobo, Japan) and prepared for subsequent RT-qPCR. RT-qPCR was performed on MyiQ2 Two-Color Real-Time qPCR Detection System (Bio-Rad, Hercules, California, USA)) under the following conditions: pre-denaturation at 95 °C for 30 s and 40 cycles of denaturation at 95 °C for 5 s, annealing at 55 °C for 10 s and extension at 72 °C for 15 s. GAPDH was employed as internal control. To control pipetting errors, each cDNA sample was run in triplicate. Relative gene expression was quantified using the ΔΔCt method [32]. The primers used in the amplification were designed by Tingke (Wuhan, Hubei, China), according to the NCBI gene database. And the detailed sequence of primers is listed in Table 1.

**Table 1:** Primers sequence for real-time reverse transcriptase polymerase chain reaction (RT-PCR)

Gene	NCBI version	Length (bp)	Sequence (5′–3′)	Product length (bp)
*GAPDH*	NM_017008.4	21	AACGACCCCTTCATTGACCTC	85
		21	CCTTGACTGTGCCGTTGAACT	
*ATG5*	NM_001014250.1	22	GTGTGAAGGAAGCTGACGCTTT	144
		21	GGAGGGTATTCCATGAGTTTC	
*BECN1*	NM_053739.2	23	AACTGGACACGAGCTTCAAGATC	78
		22	CCTGGGCTGTGGTAAGTAATGG	
*LC3B*	NM_022867.2	20	CATGCCGTCCGAGAAGACCT	70
		20	GATGAGCCGGACATCTTCCACT	
*ULK1*	NM_001108341.1	19	TGGAGGTGGCCGTCAAATG	202
		20	CGCATAGTGTGCAGGTAGTC	

### Western blotting

Chondrocytes were harvested from culture plates to obtain whole cell extracts. Briefly, the chondrocytes were rinsed with PBS, immediately exposed to RIPA Lysis Buffer (Beyotime) supplemented with 1 mM phenylmethanesulfonyl fluoride (PMSF) on ice, followed by centrifugation (12,000×*g*, 4 °C) for 20 min to clear the lysates. Total protein concentration was measured by bicinchoninic acid (BCA) protein assay (Beyotime). Aliquots containing equal amounts of protein were subjected to sodium dodecyl sulfate–polyacrylamide gel electrophoresis (SDS–PAGE); protein sample (20 μg) was loaded per lane. Samples were run on 10% gradient polyacrylamide gels (15% polyacrylamide for LC3 detection) and transferred to PVDF membranes (Millipore, USA). After transfer, the membranes were blocked with 5% bovine serum albumin (BSA) and incubated overnight with the specific primary antibodies followed by HRP-conjugated secondary antibodies. Protein detection was visualized with ECL kit (Boster) according to the manufacturer's recommendations. The protein bands were captured by the ChemiDoc XRS gel documentation system (Bio-Rad), and the intensities of bands were quantified by digital image analysis software (Quantity One, version 4.6, Bio-Rad). GAPDH quantification was used as an internal control and served to correct for variations in total protein loading.

### Transmission electron microscopy for autophagosome detection

Transmission electron microscopy (TEM) was utilized for analyzing the ultrastructural images of autophagosomes and autolysosomes. GPCs were harvested by trypsinization, washed twice with PBS and fixed with ice-cold with 2% glutaraldehyde in 0.1 M phosphate buffer. Cells were post-fixed in OsO4 and dehydrated in a graded ethanol series of acetone and then embedded for ultra-thin sectioning (Leica UC7, Germany). Stained with uranyl acetate and lead citrate, thin sections were observed by electron microscopy and photographed (Tecnai FEI EM, USA). The number of autophagic vacuoles (AVs) was counted at least from 30 cells that chosen randomly by two authors blinded to experimental condition.

### Analysis with flow cytometry (FCM)

GPCs were gently trypsinized, washed once with serum-containing medium, following washed with PBS and collected by centrifugation at 1500 rpm for 5 min. Chondrocytes were then resuspended in 500 μL binding buffer, containing 5 μL Annexin V-FITC and 10 μL PI. After that, the chondrocytes suspension was incubated in darkness for 5 min at room temperature. The suspension was analyzed using a flow cytometer (FACSort, BD Biosciences, San Jose, CA, USA), and all flow cytometry data were analyzed using FlowJo version 10.6.

### Immunofluorescent staining of LC3

In brief, GPCs were fixed with 4% paraformaldehyde in PBS for 10 min and then permeabilized with 0.1% Triton X-100 in PBS for 10 min at room temperature. Subsequently, GPCs were block with 5% BSA for 1 h and exposed to LC3 antibody in PBS with 1% BSA and 0.1% Triton X-100 over the night. After incubation with rabbit IgG Alexa fluor-594 (dilution 1:5000) at room temperature for 1 h, F-actin was labeled with Actin-tracker Green and nuclei were marked with DAPI for 5 min in the darkroom. Background fluorescence reduced after three extensive washes in PBS. Finally, fluorescence images were captured by a confocal laser scanning microscope (Zeiss, LSM 710). Images from five different fields were taken in each individual experiment. Red puncta were counted in a masked manner by two independent investigators. A total of two cells per field were counted (15 cells per experiment) and the experiment was repeated at least three times. To facilitate manual counting, the Find Edges of ImageJ were applied to process the images.

### Statistical analysis

All results are presented as the mean ± standard deviation (Mean ± SD) of three experiments. The one-way analysis of variance (ANOVA) was applied to examine differences in multiple comparisons. The post hoc test was used to assess the difference between 2 groups, while one-way ANOVA yielded significant of 0.05. Statistical analyses were performed using SPSS 17.0 statistical software (SPSS Inc., USA). The statistical significance was set as *P* < 0.05.

## Result

### CTS treatment significantly increased the mRNA levels of autophagy-related genes in GPCs

**Fig. 1:**
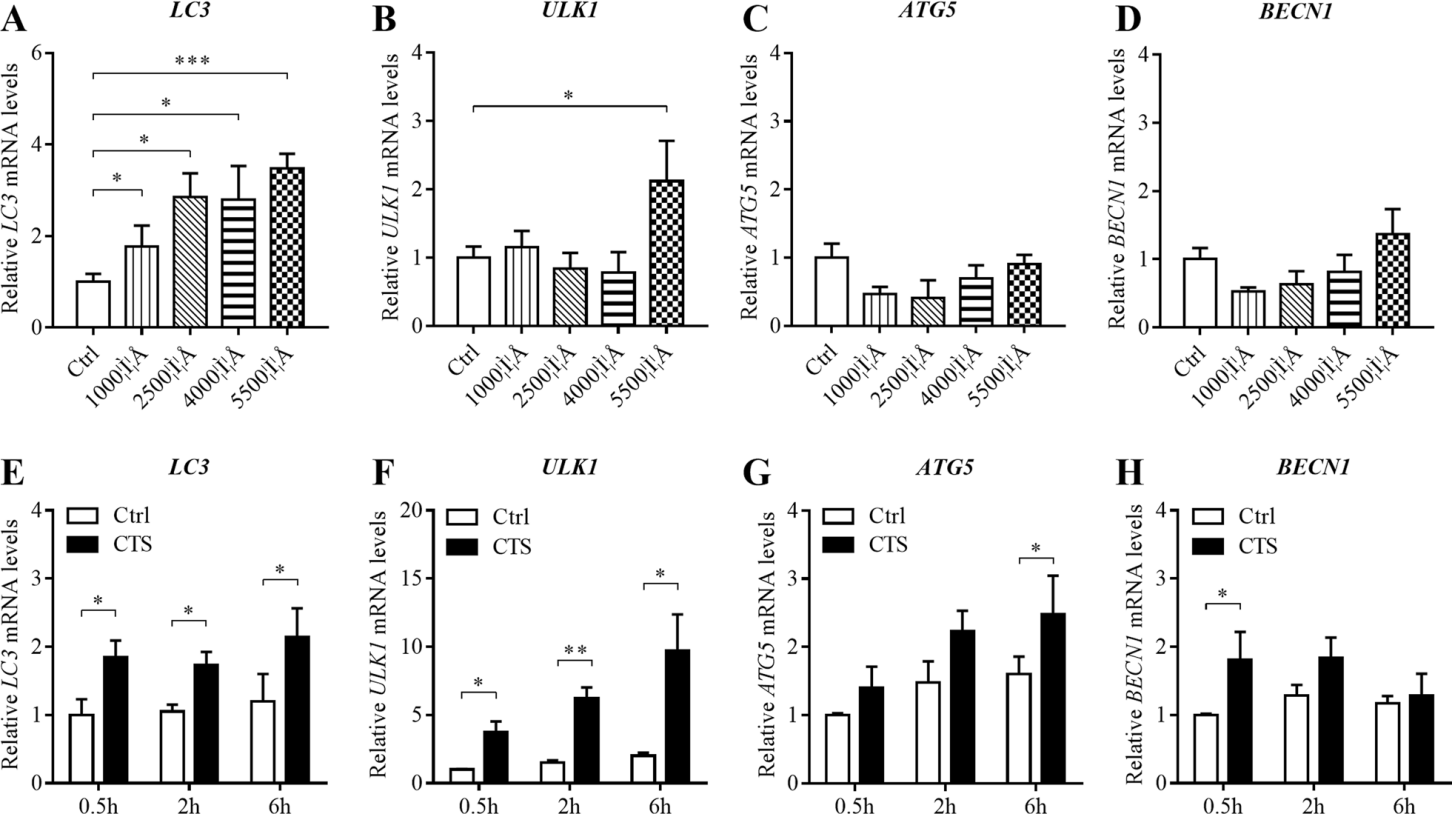
CTS treatment significantly increased the mRNA levels of autophagy-related genes in GPCs. Relative expression of *LC3, ULK1, ATG5* and *BECN1* mRNA determined by RT-qPCR. GAPDH was used as an internal control and served to normalize the results. **A**–**D** Chondrocytes were subjected to CTS at different magnitudes for 2 h. **E**–**H** Chondrocytes were subjected to CTS at 5500 με for 0.5 h, 2 h and 6 h. Error bars represent standard deviations. *n* = 3, *P < 0.05, **P < 0.01, ***P < 0.001

**Fig. 2:**
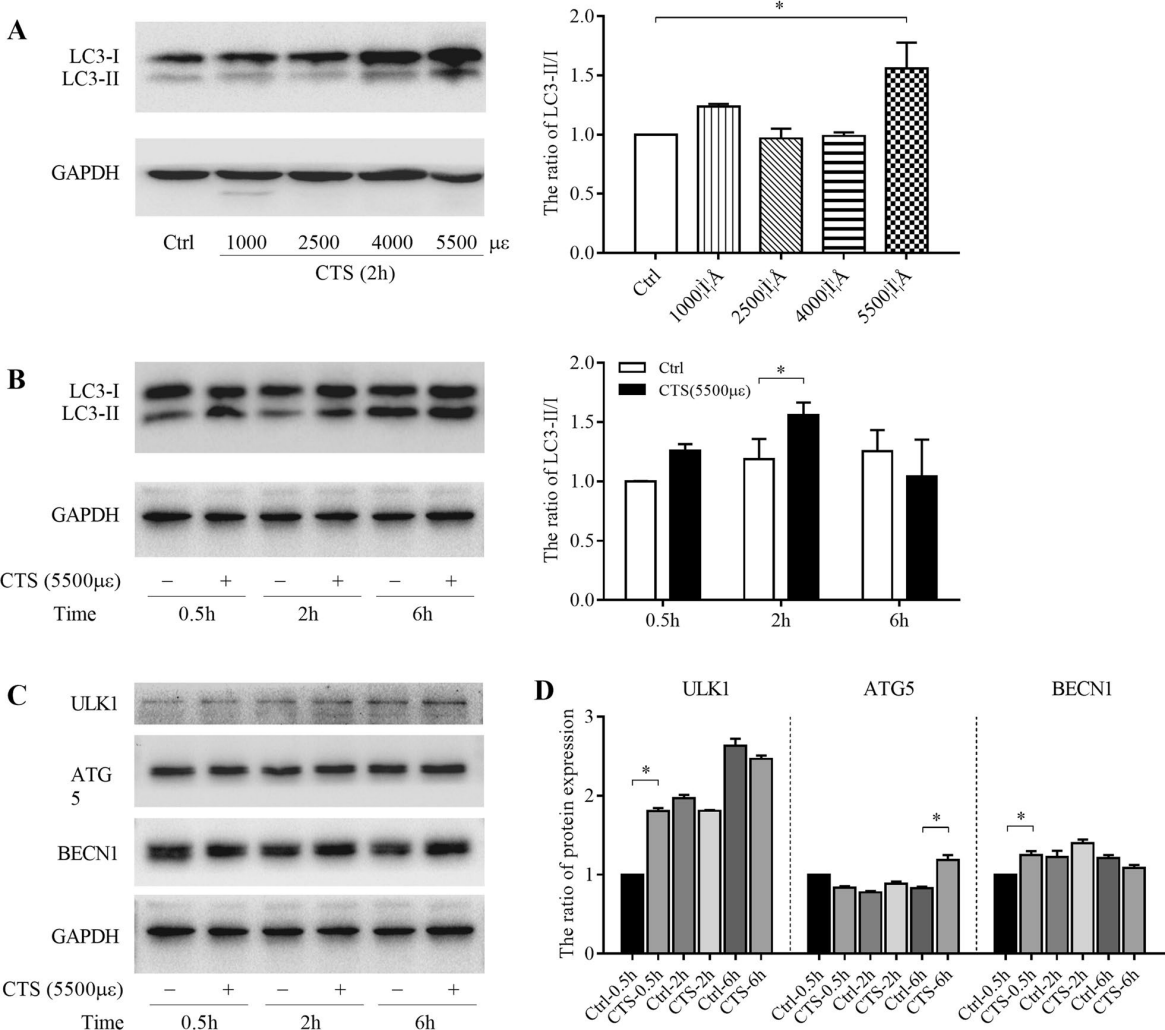
GPCs autophagy was activated by CTS in a magnitude- and time-dependent manner. The protein levels of LC3-II, LC3-I, ULK1, ATG5 and BECN1 were measured by western blotting. GAPDH was used as an internal control and served to normalize the results. **A** Chondrocytes were subjected to CTS at different magnitudes for 2 h. **B**–**D** Chondrocytes were subjected to CTS at 5500 με for 0.5 h, 2 h and 6 h. Error bars represent standard deviations. *n* = 3, *P < 0.05, **P < 0.01, ***P < 0.001

To investigate the effect of different magnitude CTS at each time point (0.5 h, 2 h and 6 h) on the expression of autophagy-related genes, the mRNA levels of *LC3, ULK1, ATG5* and *BECN1* were detected by RT-qPCR. After the 2-h stimulation, *LC3* mRNA expression in GPCs strained at 1000 με, 2500 με, 4000 με and 5500 με was significantly (*P* < 0.05) higher than control group (Fig. 1A). The mRNA level of *ULK1* in GPCs strained at 5500 με increased significantly (*P* < 0.05) compared with control group (Fig. 1B). However, the mRNA level of *BECN1* and *ATG5* showed no significant difference (*P* > 0.05, Fig. 1C, D). The results of different time points showed that *LC3* and *ULK1* mRNA expression increased significantly (*P* < 0.05) after stimulation for 0.5 h, 2 h and 6 h, compared with control group (Fig. 1E, F). After the 6-h stimulation, the mRNA level of *ATG5* was significantly increasing (*P* < 0.05, Fig. 1G). Moreover, the mRNA level of *BECN1* was significance higher than control group at 0.5 h (*P* < 0.05, Fig. 2H). CTS treatment significantly increased the mRNA levels of autophagy-related genes in GPCs, which was related to magnitude and time.

### GPCs autophagy was activated by CTS in a magnitude-dependent and time-dependent manner

LC3 protein, generally considered as a marker to monitor autophagy, is involved in the formation of phagophore when autophagy is induced. LC3-I protein is processed and modified into the PE-conjugated form, becoming LC3-II and incorporated into the extension of phagophore membrane. Sequentially, the ratio of LC3-II to LC3-I is used to detect the autophagy [33–35]. To investigate whether the increased intensity of CTS has different effects on autophagy in GPC, we stimulated GPCs with 1000 με, 2500 με, 4000 με and 5500 με tensile stress for 2 h at 2.0 Hz. There was no significant difference of the ratio of LC3-II/I in chondrocytes strained at a magnitude of 1000 με, 2000 με and 4000 με and unstrained control chondrocytes. However, it was significantly (*P* < 0.05) higher for 5500 με group than control group (Fig. 2A). To evaluate the influence of CTS on autophagy at different time points, the interventions were sustained for 0.5 h, 2 h and 6 h under 5500 με tensile stress. After treatment with CTS for 2 h, the ratio of LC3-II/I was significantly increased (*P* < 0.05) in the strained group compared with unstrained chondrocytes (Fig. 2B). Similarly, the protein levels of autophagy-related molecules, including ULK1, ATG5, and BECN1, were also upregulated in GPCs under 5500 με tensile stress at several time points (Fig. 2C, D).

### CTS did not influence GPCs apoptosis and necrosis

**Fig. 3:**
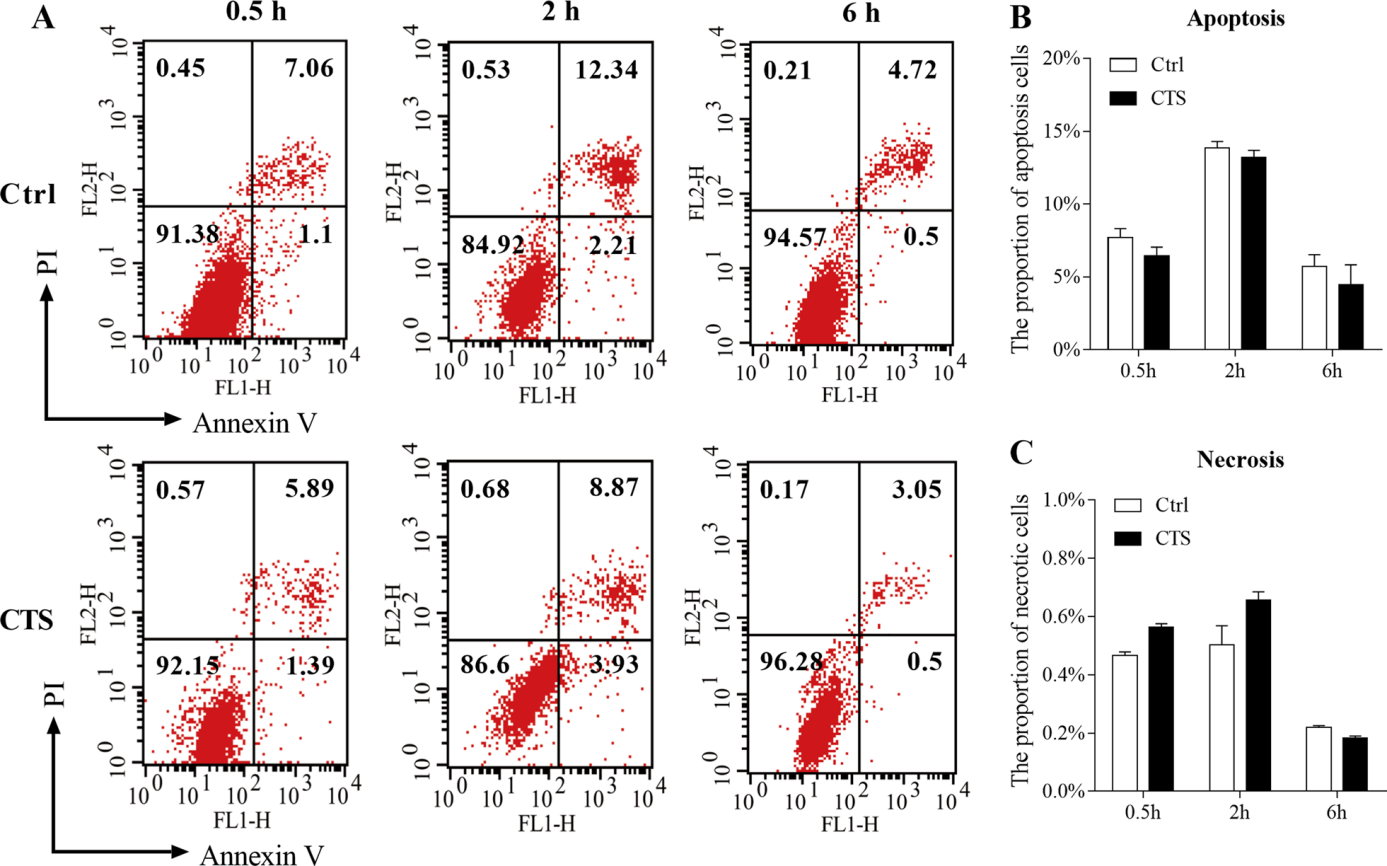
CTS had no effect on apoptosis and necrosis of GPCs. GPCs were subjected to CTS at 5500 με for 0.5 h, 2 h and 6 h. The percentage of apoptotic or necrotic chondrocytes was measured using Cell-Quest software. **A** Representative flow cytometry diagram. **B** The proportion of apoptotic chondrocytes. **C** The proportion of necrotic chondrocytes. Error bars represent standard deviations. *n* = 3

To evaluate the influence of CTS treatment on the GPCs apoptosis and necrosis, the FCM was used with PI and Annexin V. We set time points at 0.5 h, 2 h, 6 h in the condition of 5500 με CTS with 2.0 Hz. In FCM graphs (Fig. 3A), the lower left quarter indicates the living cells (Annexin V^−^/PI^−^), the upper left quarter shows necrotic cells (Annexin V^−^/PI^+^), and the quadrants of lower right and upper right represent apoptosis cells (Annexin V^+^/PI^−^ and Annexin V^+^/PI^+^). After statistical analysis, there was no significant difference between CTS group and control group at all time points (Fig. 3B, C).

### CTS promoted the autophagic vacuoles formation in GPCs

**Fig. 4:**
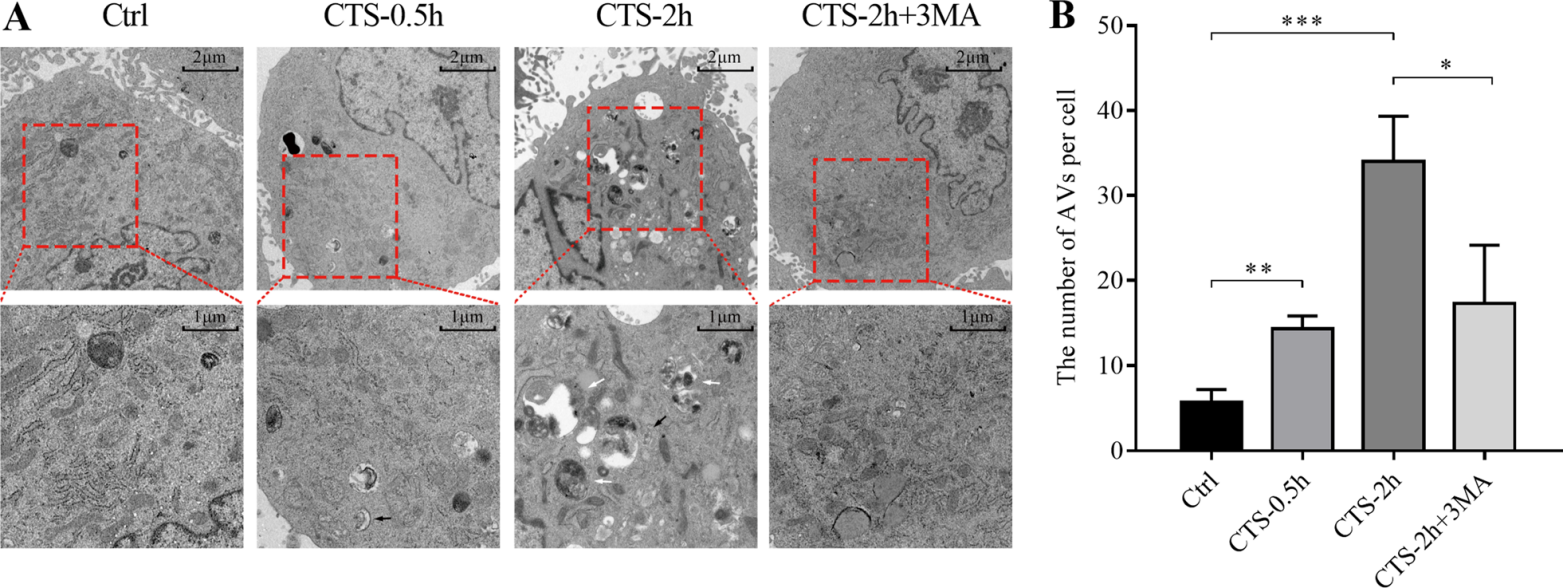
CTS promoted the formation of autophagic vacuoles in GPCs. **A** Representative images in nonstretched GPCs, GPCs received CTS (5500 με, 2.0 Hz) for 30 min, GPCs received CTS (5500 με, 2.0 Hz) for 2 h, and GPCs received CTS (5500 με, 2.0 Hz) for 2 h together with 3MA (10 mM). Black arrows indicate the autophagosome or early autophagic vacuole (AVi), and white arrows indicate the autolysosome or degradative autophagic vacuole (AVd). **B** Statistical diagram of the number of autophagic vacuoles (AVs, including AVi and AVd) in each group. Data were expressed as Mean ± SD, *n* = 3, *P < 0.05, **P < 0.005, ***P < 0.001

Since LC3-II itself is degraded by autophagy, and the amount of LC3 at a certain time point does not represent autophagy flux, there is a certain limitation of LC3-II/I ratio in estimating autophagy, and TEM was used to verify autophagosomes in GPCs [36]. GPCs received CTS of 5500 με stain with 2.0 Hz at 0.5 h and 2 h. There were some AVs in GPCs, and GPCs suffered from CTS for 0.5 h or 2 h showed an increased number of AVs (Fig. 4A, B). Additionally, we treated cells combined with CTS and autophagy inhibitor 3MA (10 mM). The results showed that the number of AVs in GPCs of the combined treatment group was significantly decreased (*P* < 0.01, Fig. 4A, B).

### CTS-induced autophagy by promoting the autophagosome formation

**Fig. 5:**
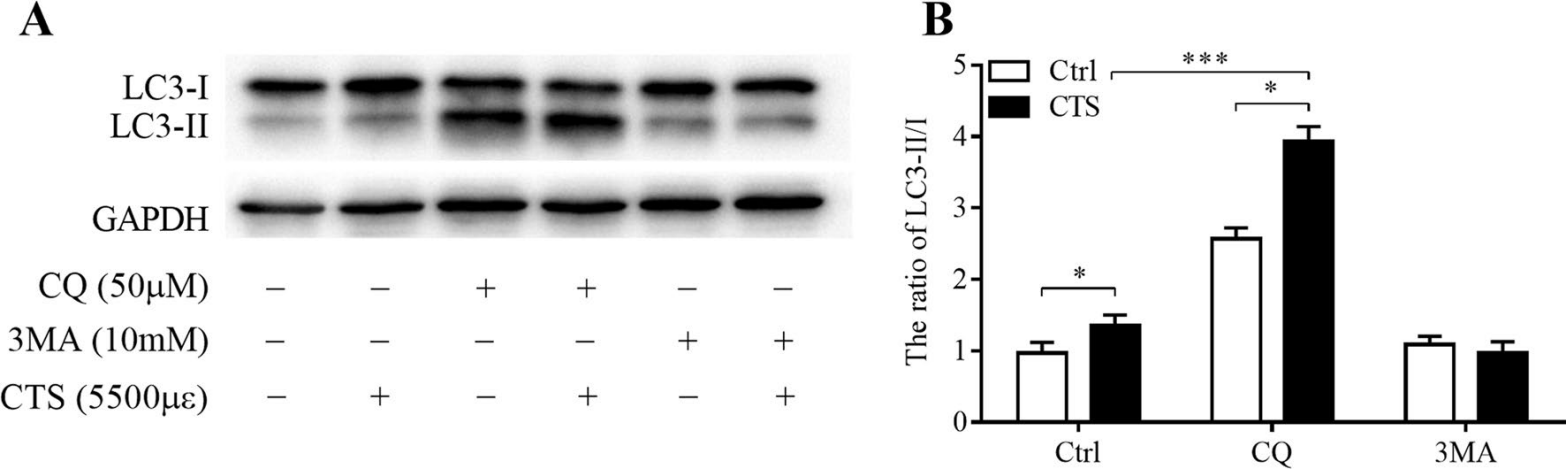
CTS-induced autophagy by promoting the autophagosomes formation. Respresentative images (**A**) and quantitative data (**B**) of LC3-II and LC3-I measured by western blotting. GAPDH was used as an internal control and served to normalize the results. GPCs were subjected to CTS (5500 με, 2.0 Hz, 2 h) with or without 3 MA and CQ. Error bars represent standard deviations. *n* = 3, *P < 0.05, ***P < 0.001

It is well known that the accumulation of autophagosomes may be due to an increase in autophagosome formation, or to a decrease in maturation and fusion with the lysosomal system. To differentiate between these two possibilities and investigate the mechanism involved in mechanical strain, 3MA (an inhibitor of the initial stages of autophagy, 10 mM) and CQ (a chemical could prevent lysosomal fusion with autophagosomes, 50 μM) were administered 1 h before stimulation [20]. Treatment with CQ did not reverse the upregulation of LC3-II/I induced by CTS (5500 με, 2 h), suggesting that CTS promoted the formation of new autophagosomes instead of interfering with lysosomal functions. However, the upregulation of the ratio of LC3-II/I could be blocked by 3MA, indicating CTS-induced GPCs autophagosome formation (Fig. 5A, B).

### F-actin cytoskeleton mediated CTS-induced autophagy

**Fig. 6:**
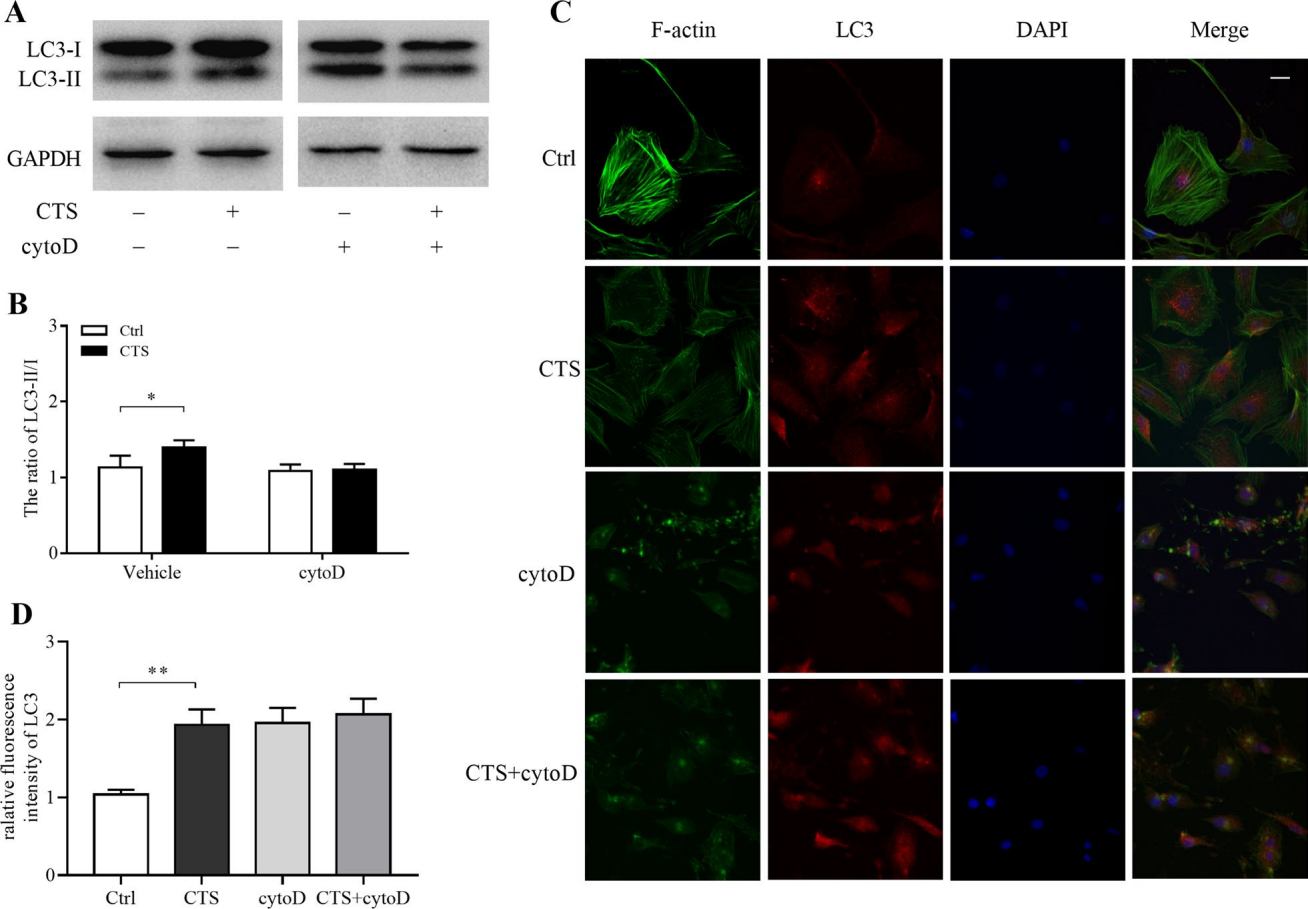
F-actin cytoskeleton mediated CTS-induced autophagy. After receiving CTS (5500 με, 2.0 Hz) for 2 h, chondrocytes were harvested for western blotting or stained with Actin-tracker (green), LC3-antibody (red) and DAPI (blue) to visualize F-actin fibers, LC3 puncta and nuclei by confocal laser scanning microscopy. **A**, **B** The protein levels of LC3-II and LC3-I were measured by western blotting. **C**, **D** Chondrocytes from control group, strained chondrocytes without cytoD treatment, unstrained chondrocytes with cytoD (1 μM) treatment and strained chondrocytes with cytoD treatment. Scale bar = 10 μm. Error bars represent standard deviations. *n* = 3, *P < 0.05, **P < 0.005

To further explore the possible mechanism of CTS-induced autophagy, the F-actin inhibitor of cytoD (1 μM, dissolved in DMSO) was used 1 h before stimulation. The ratio of LC3-II/I was detected by western blot, and there was no significant difference between CTS group and control group when cytoD participating in (Fig. 6A, B). We also measured the expression of LC3 protein by IF. After the 2-h treatment, chondrocytes were fixed and stained with Actin-tracker (green), LC3-antibody (red) and DAPI (blue) to visualize F-actin fibers, LC3 puncta and nuclei (Fig. 6C). In control chondrocytes (Ctrl group), well-organized actin fibers were observed. The shape of GPCs seemed smooth/soft and the distribution of red fluorescence (LC3) present uniformly, while the chondrocytes subjected to 5500 με CTS (CTS group) appeared sharp/rigid. In CTS chondrocytes, some red fluorescence gathered into puncta, marking the autophagosomes, around the nuclei. However, in CTS-treated chondrocytes with cytoD intervention (CTS + cytoD group), or in unstrained chondrocytes with cytoD intervention (cytoD group), it resulted in disruption of actin fiber organization and the large focal actin aggregates. Additionally, neither the fluorescence intensity nor LC3 puncta was different between CTS + cytoD group and cytoD group.

## Discussion

The four-point bending system can provide anchorage-dependent cell cyclic stress when the culture plate is bent by four metal contacts. The maximum intensity of loading is up to 7000 με and the maximal frequency is 2.0 Hz [29]. In vivo, the matrix-surrounded chondrocytes are cyclically squeezed by tensile strain, compressive stress and fluid shear stress. The aim of this study was to explore the effects of mechanical loading on the autophagy of chondrocytes, particularly while it is slightly over the physiological range.

Autophagic cell death, also known as type II programmed cell death (PCD) or autosis, has been observed since the 1960s [37]. Autophagosome formation is a very elaborate process in which cells allocate a specific set of proteins called the core autophagy machinery. The core function of autophagy machinery is supplemented by additional proteins involved in diverse cellular processes, such as membrane transport, mitochondrial and lysosomal biology. The coordination of these proteins for the autophagosome formation and degradation constitutes a highly dynamic and sophisticated response of autophagy [38]. Autophagy-related genes, conserved in higher eukaryotes, control the four steps of autophagy process: formation, elongation, maturation and fusion with the lysosomes[39]. Among the many autophagy genes, BECN1 (also termed ATG6) forms a complex with microtubule-associated protein 1 light chain 3 (also called ATG8 and LC3/MAP1LC3), which is necessary for the formation of autophagosomes [40]. The different processes of typical autophagy have been well characterized, involving the formation of different complexes of over 30 autophagy-associated proteins [41]. The ATG1-ULK complex initiates the formation of the isolation membrane (also named as phagophore), the class III phosphatidylinositol 3 kinase (PI3K) complex generates PI3-phosphate phospholipid (PI3P) for membrane biogenesis, the ATG12–ATG5–ATG16L1 complex mediates autophagosome formation and elongation, and the ATG8/LC3 lipidation system mediates closure of the autophagosome membrane. We found that the mRNA level of *LC3* was significantly upregulated with the increasing duration and magnitude of CTS treatment, compared with unstressed chondrocytes. Under the strain condition of 5500 με, the mRNA levels of *ULK1* in GPCs at different time points were significantly higher than those in the control group. Interestingly, the mRNA level *ATG5* and *BECN1* were significantly higher than control group at the magnitude of 5500 με for 6 h and 0.5 h, respectively.

Some researchers query that the increased ATGs mRNA levels are not enough to verify the occurrence of autophagy. They believe that the transformation of cytosolic type of LC3 (LC3-I) into membrane type (LC3-II) is a more reliable indicator of autophagy [42]. During autophagy, LC3-I binds with phosphatidylethanolamine to form LC3-II. LC3-II is a protein located on the membrane of autophagosomes and participates in the extension of autophagosome membrane. The ratio of LC3-II to LC3-I is usually used to evaluate the autophagy flux by western blotting. Thus, we detected the ratio of LC3-II/I on besides investigating the level of ATGs mRNA. The results showed that low intensity of tensile strain did not affect the level of autophagy, but high tensile strain (5500 με) induced GPCs autophagy activation. The results showed that compared with control group, the ratio of LC3-II/I of growth plate chondrocytes was slightly increased after 0.5 h of CTS treatment. After 2 h, the ratio between the two groups increased significantly, but after 6 h, there was no significant difference. In combination with the prerequisites of autophagy, we speculated the reason was that stress signal was transferred to cells from the external mechanical environment, leading to the increase of autophagy level at the early stage of mechanical loading. When the mechanical loading continues to exist, GPCs adapt to the external environment, and the loading that is as the same as the initial condition is not enough to be sensitive to the “stress signal,” so it would not activate the autophagy process. King et al. stimulated MDA-MB-231 cells with 0.25 kPa compression and found that autophagy occurred in the first 30 min and reverted to base level 90 min later [19]. Porter et al. found that static mechanical stress (20% elongation) increased the level of LC3-II in trabecular meshwork cells from 30 min, and the level of Atg5 and Atg12 decreased after 16-h stretching culture [43]. Moreover, Xu et al. treated the end-plate chondrocytes with a 10% elongation CTS at 0.5 Hz, and autophagy was significantly activated in the 5-day group but decreased in the 10- and 20-day groups [44]. The results of these studies are similar to ours, which support our conclusions. And it is a very interesting point and will be one of our follow-up studies.

Autophagy is accompanied by a series of changes of cell ultrastructural components. TEM is important method for qualitative and quantitative analysis of autophagy, and has been regarded as the gold standard for observing autophagy [42]. The typical autophagosome (also referred to as initial autophagic vacuoles, AVi) has a double membrane that is usually visible at least partly as two parallel membrane bilayers separated by an electron-lucent cleft. The autophagosome engulfs decrepit organelles and is transferred to the degradative lysosome, followed by inner membrane degraded and constituent components recycled or exported, in which period we name it as autolysosome. Autolysosome (or named degradative autophagic vacuoles, AVd) usually has only one limiting membrane, containing cytoplasmic material and/or organelles at various stages of degradation. And it can sequester the cytoplasm, long-lived proteins and protein aggregates, defective organelles and even viruses or bacteria [38]. In this study, we observed GPCs autophagosomes by TEM and found that CTS can promote the autophagosome formation, demonstrating that autophagy can be induced by CTS.

Autophagy, apoptosis and necrosis are different ways of cell death, and there are difference and similarity among them. Autophagy and apoptosis both belong to programmed cell death, and there are many connections when they perform biological functions under physiological and pathological conditions. It reports that autophagy and apoptosis can be mutually inhibited and triggered [45]. Flow cytometry analysis showed that there were no significant differences in apoptosis and necrosis of chondrocytes between the experimental group and the control group after 0.5 h, 2 h and 6 h of stress, indicating that the autophagy activation induced by CTS was not secondary to apoptosis and necrosis.

The accumulation of autophagosomes can be attributed to the formation as well as the degradation. 3MA is a selective inhibitor of PI3K signaling pathway, which can inhibit the formation of autophagosomes [20]. The results of this study showed that the CTS-induced autophagy was inhibited by 3-MA, suggesting that the PI3K signaling pathway was involved in CTS-induced autophagy activation of GPCs. CQ is another autophagy inhibitor that inhibits lysosomal degradation of autophagosomes. The results showed that LC3-II protein was significantly accumulated after CQ treatment. However, the ratio of LC3-II to LC3-I of CTS group was still distinctly higher than control group. The findings suggested that CTS can increase the level of GPCs autophagy by promoting the formation of autophagosomes rather than by inhibiting the accumulation of degraded autophagosomes, which is similar to Memert’s and Zheng’s studies [34, 46].

F-actin is an important molecule for cellular reception and transmission of mechanical signals [47]. Several researchers have reported that the cytoskeleton integrity can affect the level of autophagy [41, 48]. Zhuo et al. observed that autophagosomes accumulate in the presence of cytoD, and the formation of AVd is blocked under both nutrient-rich and starvation conditions [28]. Lee et al. found that F-actin is a selective requirement for basic autophagy rather than starvation-induced autophagy, and the actin cytoskeleton is required for the final step of basic autophagy: fusion of autophagosomes and lysosomes [27]. In addition, M.O. Aguilera et al. reported that F-actin can promote the formation of PI3P and further form autophagosomes, and the increasing of AVs was abolished under starvation condition when the actin cytoskeleton is depolymerized, but the maturation of remaining autophagosomes was not affected [49]. In the present study, we found that the ratio of LC3-II to LC3-I has no difference between CTS groups and control group when cytoD has been added, suggesting that the depolymerization of F-actin can inhibit the autophagy induced by CTS. At the same time, whether received CTS or not, the increased level of autophagy after cytoD treatment indicated that F-actin may not only be involved in the transduction of mechanical signals to promote the formation of autophagosomes, but also participate in the process that autophagosomes transfer to lysosome. Although GFP-LC3 or mRFP-GFP-LC3 is useful for monitoring autophagy in vitro and in vivo, immunostaining anti-LC3/Atg8 antibodies have the advantage of detecting endogenous protein and avoiding potential interference due to overexpression [42]. Confocal laser scan microscopy showed that after CTS intervention, chondrocytes become sharp and stiff, and red fluorescence aggregated into spots in the cytoplasm, suggesting the accumulation of autophagosomes. The intensity and number of red puncta had no significant difference between CTS groups and control group after treated by cytoD, further suggesting that F-actin mediates the formation of AVs induced by CTS.

Previous study has suggested that mechanical forces rapidly induce a proportional stiffening of the cytoskeletal cortex [50]. According to the results above, the level of autophagy first increased and then decreased under the intervention of CTS, which can be inferred that the promotion of autophagy is mediated by F-actin in the early stage. And the level of autophagy will not increase when F-actin reconstructed and adapted to the mechanical loading. Other study also indicated that by mechanical force-induced autophagy is transient, typically about 90 min, and lasts until the cells remodel their cortex to relieve stress [51]. This phenomenon is in line with most researchers’ understanding of the physiological effect of autophagy [43, 52]: before cytoskeleton adapting to forces or elevated pressure, the autophagy of GPCs was increasing to safeguard themselves and affect metabolic processes (including the synthesis of collagen and proteoglycan) to help rebalance cellular and tissue function. Eventually, some cells made it through the harsh environment, while others still had trouble adapting to the stress and progressed toward autophagy death. Since then, F-action may no longer transmit signal of tensile force to downstream signaling pathways, and autophagy levels of surviving GPCs returned to basal levels after prolonged mechanical stimulation. It is reasonable to assume that the dysregulation of autophagy would trigger GPCs death and lead to diseases if mechanical forces exceed the physiological range.

Increasing evidence suggests that autophagy is a self-protective mechanism in cartilage which maintains homeostasis, as well as cellular response to different types of stress [53–55]. The development of safe and effective methods that can enhance autophagic activity or restore autophagy flux is a promising strategy for the treatment of cartilage damage [56–60]. This study suggested that CTS with a frequency of 2.0 Hz, magnitude of 5500 με and duration of 2 h was appropriate condition for inducing autophagy activation of GPCs, providing biomechanical parameters for further research.

## Conclusion

In summary, our study suggests that high-intensity CTS can induce autophagy in GPCs, and cytoskeletal F-actin microfilaments organization plays a key role in it. This provides a new sight that CTS may maintain GPCs homeostasis by mediating autophagy.

## Data Availability

All data generated or analyzed during this study are included in this published article.

## Abbreviations

CTS: Cyclic tensile stress
GPCs: Growth plate chondrocytes
FACS: Fluorescence-activated cell sorting
3MA: 3-Methyladenine
CQ: Chloroquine
cytoD: Cytochalasin D
ECM: Extracellular matrix
G-actin: Globular actin
F-actin: Filamentous actin
LC3A/B: Microtubule-associated protein 1 light chain 3 A/B
ATG5: Autophagy-related gene 5
BECN1: Beclin-1
Bcl-2: B-cell lymphoma-2
Ulk-1: Unc-51-like autophagy-activating kinase 1
SQSTM1/p62: Sequestosome 1/p62
HRP: Horseradish peroxidase
IF: Immunofluorescence
PI: Propidium iodide
PBS: Phosphate-buffered saline
DAPI: 4',6-Diamidino-2-phenylindole dihydrochloride
DMEM/F-12: Dulbecco's modified Eagle's medium/Ham's F-12
FBS: Fetal bovine serum
RT-qPCR: Reverse transcription-quantitative polymerase chain reaction
PMSF: Phenylmethanesulfonyl fluoride
BCA: Bicinchoninic acid
SDS–PAGE: Sodium dodecyl sulfate–polyacrylamide gel electrophoresis
BSA: Bovine serum albumin
TEM: Transmission electron microscopy
FCM: Analysis with flow cytometry
ANOVA: One-way analysis of variance
AVs: Autophagic vacuoles
PCD: Programmed cell death
PI3K: Phosphatidylinositol 3 kinase
PI3P: PI3-phosphate phospholipid
